# The International Congress on Tuberculosis

**Published:** 1908-09

**Authors:** 


### EDITORIAL DEPARTMENT.

### THE INTERNATIONAL CONGRESS ON TUBERCULOSIS, WASHINGTON, D. C., SEPTEMBER 21-OCTOBER 12, 1908.

In conformity to a resolution passed by the Texas State Medical Association, at its last annual meeting, Dr. H. W. Cummings, President, has selected quite a list of the prominent physicians of the State to represent the Association at the International Congress on Tuberculosis, which meets in Washington, D. C., September 21 to October 12, next.

Looking upon this meeting as one of vast importance to the profession, as well as the public, Dr. Cummings has made every effort to select men especially interested in this great movement, as well as who would strive to attend the meeting.

He has sought the aid of the officers of the Association that he might not overlook any who desired to avail themselves of this opportunity to help in the advancement of the great fight against tuberculosis, as well as gain such knowledge as this meeting will afford.

The doctor is very much pleased with the prospects of a large attendance as the replies to his appointments show a large per cent of acceptances. It is, however, his desire to add others still to the list, and he requests us to ask any member of the State Association who would like to attend to notify him, when he will take pleasure in sending credentials.

With the delegates to be appointed by State Health Officer Brumby from the Health Officers' Association, and those from other State organizations, Texas is expected to have the largest delegation in the Union.

The interest manifested in this Congress by the many prominent business men and leading women of the State is very gratifying to those who have taken a lead in the work of interesting Texas in this meeting.

The public is beginning to appreciate the efforts of the medical profession in their fight for a better system of public health, and when the citizenship of our State more fully realize the importance of preventative medicine then, and not before, will their co-operation be secured in the enactment and enforcement of the proper sanitary laws so much needed in Texas today.

Dr. Cummings urges every delegate to attend this meeting that he may return with a store of information and enthusiasm which will help arouse Texas to a determined fight against this dread malady.

The list below are named as delegates:

Dr. F. E. Daniel, Austin; Dr. J. M. Inge, Denton; Dr. A. Garwood, New Braunfels; Dr. J. H. Wood, Hubbard; Dr. I. C. Chase, Fort Worth; Dr. S. C. Red, Houston; Dr. J. S. Lankford, San Antonio; Dr. W. E. Sturgis, Stephenville; Dr. C. E. Cantrell, Greenville; Dr. C. A. Smith, Texarkana; Dr. S. T. Turner, El Paso; Dr. N. J. Phenix, Colorado; Dr. D. R. Fly, Amarillo; Dr. W. B. Russ, San Antonio; Dr. H. J. Hamilton, Laredo; Dr. J. C. Anderson, Granger; Dr. W. A. McCamly, Wharton; Dr. John T. Moore, Galveston; Dr. Jas. A. Hill, Groveton; Dr. Holman Taylor, Marshall; Dr. J. A. Holloway, Round Rock; Dr. W. F. Gallagher, El Paso; Dr. D. H. Huffaker, El Paso; Dr. Chas. M. Hendricks, El Paso; Dr. G. N. Thomas, El Paso; Dr. W. J. Vinsant, El Paso; Dr. J. W. Warren, Snyder; Dr. W. D. Patton, Amarillo; Dr. D. H. Barnes, Tulia; Dr. J. A. Hedrick, Dalhart; Dr. J. E. Dotson, Vernon; Dr. J. W. Albert, Childress; Dr. C. M. Alexander, Coleman; Dr. Thos. Dorbandt, Lampasas; Dr. Boyd Cormick, San Angelo; Dr. F. B. Magruda, San Angelo; Dr. Newton Long, Santa Anna; Dr. F. Paschal, San Antonio; Dr. Theo. Y. Hull, San Antonio; Dr. W. A. King, San Antonio; Dr. C. C. Jones, Comfort; Dr. D. Y. Willbern, Runge; Dr. E. E. Palmer, Kerrville; Dr. Fred J. Combe, Brownsville; Dr. C. A. Grey, Bonham; Dr. M. M. Smith, Dallas; Dr. Henry Redmond, Corpus Christi; Dr. E. A. Spohn, Corpus Christi; Dr. Frank D. Boyd, Fort Worth; Dr. W. A. Duringer, Fort Worth; Dr. C. P. Yeager; Corpus Christi; Dr. T. J. Turpin, Corpus Christi; Dr. H. G. Heaney, Corpus Christi; Dr. E. H. Sauvignet, Laredo; Dr. L. L. Lacy, Austin; Dr. T. J. Bennett, Austin; Dr. Walter Shropshire, Yoakum; Dr. F. O. Norris, Eagle Lake; Dr. F. Kirrum, Cuero; Dr. G. W. Allen, Flatonia; Dr. F. B. Shields, Victoria; Dr. John T. Moore, Galveston; Dr. David H. Lawrence, Galveston; Dr. W. S. Carter, Galveston; Dr. Edward Randall, Galveston; Dr. J. E. Thompson, Galveston; Dr. J. M. O'Farrell, Richmond; Dr. J. W. Scott, Houston; Dr. Joe Stewart, Houston; Dr. Joseph Mullen, Houston; Dr. R. E. B. Bledsoe, Somerville; Dr. Geo. R. Barnes,

Trinity; Dr. Albert Woldert, Tyler; Dr. W. G. Jameson, Palestine; Dr. W. G. Lipscomb, Crockett; Dr. M. L. Graves, Galveston; Dr. Alex Spever, Temple; Dr. W. L. Crosthwait, Holland; Dr. J. M. Cutchen, Waco; Dr. G. B. Foscue, Waco; Dr. M. L. Langford, Mart; Dr. J. H. Bernett, Kopperl; Dr. Geo. F. Perry, Hamilton; Dr. J. H. Petty, Franklin; Dr. W. H. Blythe, Mount Pleasant; Dr. Geo. H. Tabor, Dallas; Dr. A. W. Carnes, Hutchins; Dr. T. J. Wilson, Sherman; Dr. B. F. Calhoun, Beaumont; Dr. A. B. Small, Dallas; Dr. R. H. Gough, Hillsboro; Dr. J. H. McCracken, Mineral Wells; Dr. J. D. Osborne, Cleburne; Dr. Edward H. Cary, Dallas; Dr. J. B. Shelmire, Dallas; Dr. C. M. Rosser, Dallas; Dr. W. R. Blailock, Dallas; Dr. Henry K. Leake, Dallas; Dr. Bacon Saunders, Fort Worth; Dr. W. H. Allen, Marlin; Dr. Daniel Parker, Calvert; Dr. K. H. Aynesworth, Waco; Dr. H. M. Lanham, Waco; Dr. W. T. West, Waxahachie; Dr. W. C. Blalock, Kosse; Dr. J. W. McLaughlin, Austin; Dr. A. L. Norsworthy, Houston; Dr. Geo. H. Lee, Galveston; Dr. F. U. Painter, Pilot Point; Dr. J. W. Torbett, Marlin; Dr. J. Phillip Gibbs, Houston; Dr. T. B. Kittrell, Texarkana; Dr. P. E. Parker, Bay City; Dr. P. W. Pearson, Emory; Dr. B. F. Stout, San Antonio; Dr. Pierre Wilson, Dallas; Dr. W. M. Wier, Houston; Dr. H. L. Warwick, Fort Worth; Dr. J. S. Wilkins, Wellington; Dr. W. B. Weber, Round Rock; Dr. Geo. H. Moody, San Antonio; Dr. Russell Caffery, San Antonio; Dr. Jas. H. Bindley, San Antonio; Dr. W. E. Wright, San Antonio; Dr. A. D. Du Pay, San Antonio; Dr. Chas. W. Adgen, San Antonio; Hon. R. L. Ball, San Antonio; Prof. Wesley Peacock, San Antonio; Mr. George W. Breckenridge, San Antonio; Rev. John T. Nicholson, Houston; Mrs. Emma H. Townsend, Corsicana; Dr. Thomas J. Farmer, Dublin; Dr. F. D. Garrett, Gainesville; Dr. P. S. Russett, Batson; Dr. D. S. Blair, Klondike; Dr. Jas. H. Taylor, Marshall.

